# Theoretical calculations of formation and reactivity of *o*-quinomethide derivatives of resorcin[4]arene with reference to empirical data

**DOI:** 10.1098/rsos.220541

**Published:** 2022-10-12

**Authors:** Waldemar Iwanek

**Affiliations:** Faculty of Chemical Technology and Engineering, Bydgoszcz University of Science and Technology, Seminaryjna 3, 85-326 Bydgoszcz, Poland

**Keywords:** resorcin[4]arene, *o*-quinone methide, DFT calculations

## Abstract

This paper describes theoretical reaction pathways of alkoxybenzyl derivatives of resorcin[4]arene leading to the formation of *o*-quinomethide derivatives of resorcin[4]arene (*o-*QMR[4]A). For each case, the activation energies for the formation of one *o*-QMR[4]A unit and the activation energies for the backward reaction were calculated. Based on the calculated reaction pathways, the reaction mechanism of *o*-QMR[4]A formation was proposed. Using the example of *o*-QMR[4]A generated from a methoxy derivative of resorcin[4]arene, the activation energies with selected nucleophiles were calculated and the reaction mechanisms discussed. Reaction path calculations were performed using the nudged elastic band method and semiempirical extended tight-binding method (GFN2-xTB). Using hydroxybenzyl derivatives of resorcin[4]arene as an example, a comparison of calculated activation energies by selected density-functional theory methods with GFN2-xTB and B97-3c geometries was performed. B97-3c and wB97XD methods were used to calculate the energies of the reactants (R), transition states (TS) and products (P) of the analysed reactions. Theoretical reaction mechanisms were discussed with respect to the orbital-weighted Fukui dual descriptor (Δ*f_w_*) and experimental data.

## 1. Introduction

*Ortho*-quinone methides (*o*-QMs) are short-lived, highly reactive and versatile intermediates in organic synthesis, material chemistry, fine chemicals and pharmaceuticals [1–3]. They are generally formed by the reaction of phenols with aldehyde in the presence of an acid or a base [4]. They react very rapidly with nucleophiles and undergo efficient Diels-Alder reactions with electron-rich olefins [5]. *o*-QMs containing an additional double-bond spontaneous transform via oxa-6π electrocyclization to *2H*-benzopyrans products. Expansive reactivity of the *o*-QM can be used in the reaction for the construction of various natural products [6–8].

In several recent works, the authors showed a possibility of generating *o*-quinomethide derivatives of resorcin[4]arene (*o*-QMR[4]A), especially from methoxy derivatives of resorcin[4]arene and using their reactivity to form new derivatives of resorcin[4]arene. Their reactions with nucleophiles containing active hydrogen (HNu) [9], nucleophiles without active hydrogen (Nu) [10] and also with CH-acids [11–13] and compounds containing a double bond [14–16] were studied. About the fact that these reactions run through o-QMR[4]A as an intermediate product, the authors concluded from the structure of the obtained products and literature data on the reactivity of phenol derivatives [17].

Resorcin[4]arenes are macrocyclic molecules that are widely used in supramolecular chemistry [18]. They often contain over 150 atoms per molecule. For this reason, the calculation of theoretical reaction paths by accurate density-functional theory (DFT) methods is very limited and computationally expensive. This situation changed after Grimme's team introduced fast semiempirical quantum-mechanical methods (extended tight binding) for geometry optimization called GFNn-xTB methods [19,20]. Because of their speed, exceeding more than 1000 times the speed of the fastest DFT methods, they offer new perspectives for the rapid study of reaction mechanisms. Furthermore, the GFNn-xTB methods provide reasonably accurate transition state (TS) geometries, thus enabling more efficient energy optimization at a higher level of theory [21,22]. These advantages of GFNn-xTB methods provide theoretical insight into the course of reactions involving macrocyclic compounds. The purpose of the study was to: (i) compare of the activation energy of the *o*-QMR[4]A generation reaction with hydroxybenzyl derivatives of resorcin[4]arene (HBR[4]A) with selected DFT methods on the GFN2-xTB and B97-3c [23] geometries; (ii) theoretically examinate the possibility of generating *o-*QMR[4]A from alkoxybenzyl derivatives of resorcin[4]arene (ABR[4]A); (iii) calculate and compare the activation energies of *o*-QMR[4]A reactions with selected nucleophiles; and (iv) better understanding the reaction mechanisms.

## 2. Results

For theoretical calculations of the activation energy of the formation of *o*-QMR[4]A, the ABR[4]A and the HBR[4]A were chosen. Owing to good solubility of these derivatives in chloroform, all theoretical calculations of *o-*QMR[4]A formation were carried out in chloroform. Authors were particularly keen to use the methoxy benzyl derivative of resorcin[4]arene in experimental work because of its ease of synthesis and purification by crystallization. Moreover, the departing methanol molecule during thermal activation most often passes into the gas phase under the reaction conditions and does not compete with other reactants with a higher boiling point.

### 2.1. Calculation procedure

**Scheme 1.**
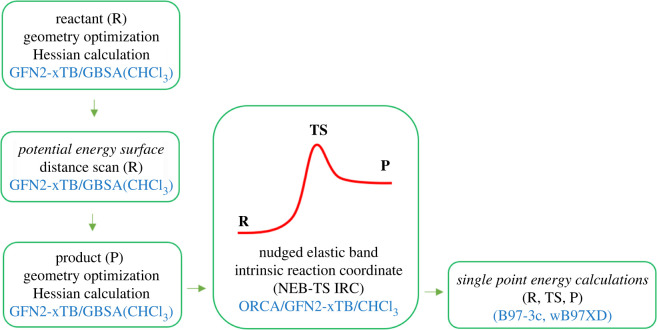
Calculation procedure of reaction pathways and energies of reactants (R), transition states (TS) and products (P) for HBR[4]A and ABR[4]A.

Theoretical calculations of reaction pathways and activation energies of forward ($\Delta E_{a}^{F}$) and backward ($\Delta E_{a}^{B}$) reactions were carried out for only one resorcinol unit in HBR[4]A and ABR[4]A. Calculations were started by optimizing the structure of HBR[4]A and ABR[4]A in chloroform with semiempirical extended tight-binding method (GFN2-xTB v.6.32). Calculations were carried out at the ‘normal’ level of precision using ‘GBSA’ as a solvent model (generalized Born (GB) model with surface area (SA) contributions [24]) in chloroform. For each reactant (R), after geometry optimization, Hessian calculations were performed to verify the infrared (IR) spectrum (lack of imaginary modes). Next, a scan of the reactant potential energy surface (PES) was performed as a function of the distance between the carbon of the benzyl group and the oxygen atom of the alkoxy or hydroxyl group (H_2_C-OR(H)). Scanning was started from the equilibrium binding distance (RW) with a step of 0.1 nm to the RW + 2 nm distance. Subsequently, optimization calculations were performed on the geometry of the resulting product (P) and the Hessian using the GFN2-xTB method with parameters as before. After optimizing the geometry of the reactant and product structures, a TS search was performed using the nudged elastic band (NEB) method [25]. The search was carried out using Orca 4.2.1 software [26] implemented with GFNn-xTB methods. For each case, 25 scans of the transition path from reactants to products were performed using the ‘NEB-TS’ command. After the TS search was completed, its Hessian was calculated in order to verify that the calculated IR spectrum has only one negative (imaginary) frequency. To confirm that the localized TS lies on the minimum energy path between two assumed minima (reactant, product), calculations were performed using the additional intrinsic reaction coordinate (IRC) method [27], which was obtained by using the ‘NEB-TS IRC’ command in the Orca program. Calculations were performed in chloroform using a solvation model based on density as a solvent model [28]. For the optimized R, TS and P structures, single-point energy (SPE) calculations in chloroform using the conductor-like polarizable continuum solvent model [29] were performed. The DFT method B97-3c using Orca 4.2.1 and wB97XD using the Gaussian16 package [30] were applied. The general procedure for conducting the calculations is shown in scheme 1.

### 2.2. Generation of *o*-QMR[4]A from HBR[4]A and ABR[4]A and reaction mechanism

**Figure 1.**
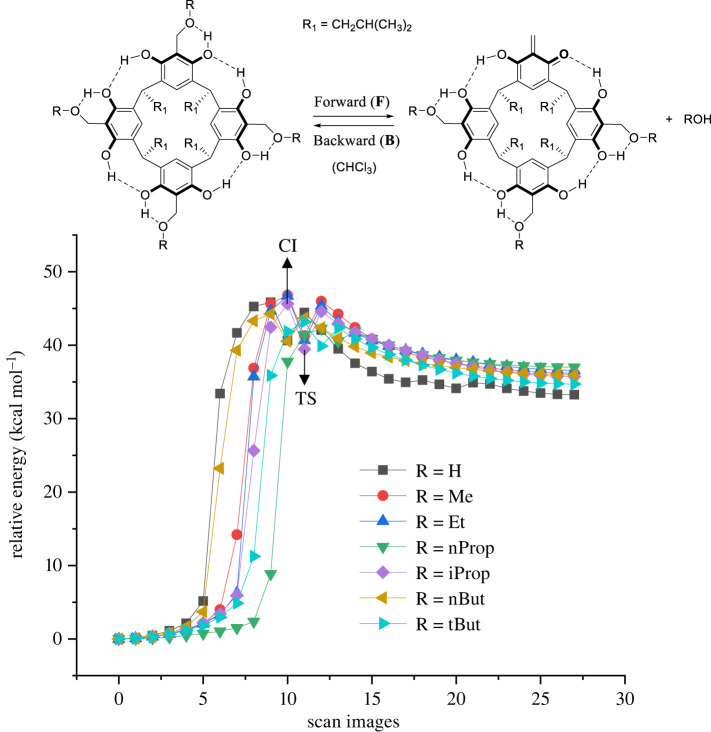
Relative energy changes as a function of number of scans of reaction paths of the *o*-QMR[4]A generated from HBR[4]A and ABR[4]A performed using the NEB-TS IRC method. TS, transition state; CI, climbing-image.

Figure 1 shows the relative energy dependence of the reaction pathway corresponding to each scan on the path from reactants to products for ABR[4]A and HBR[4]A. For the isopropoxy-benzyl derivative of resorcin[4]arene, TS and climbing-image (CI) were marked. The observed reaction pathways for the generation of *o-*QMR[4]A from HBR[4]A and ABR[4]A derivatives are very similar. The only difference is that depending on the type of derivative, TS is achieved with a slightly different number of scans. This means that, for example, the TS for the HBR[4]A is reached faster than for the n-propyloxy benzyl derivative of resorcin[4]arene. For HBR[4]A, several DFT methods were used to calculate the activation energy of *o-*QMR[4]A formation and its backward reaction. This allowed information to be obtained on the discrepancy between the obtained activation energies using the GFN2-xTB method and the DFT methods, as well as on the DFT methods themselves. The selection of DFT methods was based on works discussing the accuracy of DFT methods located at different levels of Jacob's ladder [31]. The Jacob's ladder hierarchy is commonly used to classify DFT methods.

Computing high-level SPEs on lower level structures is standard practice in computational chemistry to significantly reduce computation times. Calculations of SPE with DFT methods on GFN2-xTB geometries presented in [32] have given very reasonable results. This approach is computationally much cheaper than the full geometry optimization by the B97-3c method for resorcin[4]arene derivatives. For example, the reagent geometry optimization for HBR[4]A using Orca 4.2.1 software and a laptop equipped with an i5–7300H processor are 3 min (GFN2-xTB) versus 700 min (B97-3c), respectively. In the case of TS for HBR[4]A, the geometry optimization by the B97-3c method is significantly longer and amounts to 4800 min. However, for comparison purposes, SPE calculations by DFT methods were performed on HBR[4]A geometries calculated by B97-3c method. The *ω*B97M-D3BJ method was chosen as a reference, which offers a very good compromise between the efficiency and calculation accuracy, and it accuracy can be comparable to the double hybrid [33].

Two generalized gradient approximation (GGA) methods (B97-3c and revPBE-D4 [34]), two meta-GGA methods (B97M-D4 [35] and B97M-V [36]), differing by corrections for the contribution of dispersion in the calculated energy, and three hybrid DFT methods (B3LYP-D4 [37], wB97XD and wB97M-D3BJ) were chosen to estimate the magnitude of the activation energy of reactions involving HBR[4]A. For all DFT methods (except B97-3c), the def2-TZVP orbitals function base was used. The B97-3c composite method for fast DFT calculations contains the minimal base of mTZVP orbitals. The activation energies of the forward reaction of *o*-QMR[4]A from HBR[4]A calculated by DFT methods ($\Delta E_{a}^{F}$) and the activation energies of the backward reaction ($\Delta E_{a}^{B}$) based on the geometries calculated by GNF2-xTB and B97-3c methods are summarized in table 1.

**Table 1.** Activation energies of the forward reaction of *o*-QMR[4]A from HBR[4]A ($\Delta E_{a}^{F}$) and activation energies of the backward reaction ($\Delta E_{a}^{B}$) calculated by selected DFT methods based on GFN2-xTB and B97-3c geometries.

R = H	GGA		meta-GGA		hybrid		
GFN2-xTB							
	B97-3c	revPBE-D4	B97M-D4	B97M-V	B3LYP-D4	wB97XD	wB97M-D3BJ
$\Delta E_{a}^{F}$/(kcal mol^−1^)	22.26	22.08	25.93	26.30	26.22	32.29	31.10
$\Delta E_{a}^{B}$ (kcal mol^−1^)	9.64	8.65	12.59	11.80	13.11	13.92	13.25
B97-3c							
$\Delta E_{a}^{F}$/(kcal mol^−1^)	22.74	22.07	26.91	27.14	26.86	32.82	31.43
$\Delta E_{a}^{B}$ (kcal mol^−1^)	10.49	9.16	13.83	12.92	13.63	14.22	13.68

The conclusions drawn from the data of table 1 are as follows: (i) the activation energies calculated by DFT methods for the geometries calculated by the GFN2-xTB and B97-3c methods agree very well; (ii) the differences in the calculated activation energies by DFT methods located on different rungs of the Jacob's ladder reach values of about 4–5 kcal mol^−1^ between rungs (except for B3LYP-D4, which is closing to meta-GGA methods), which is in agreement with literature data [38].

The methods most compatible with the calculated values of activation energies, and recommended especially for the description of hydrogen bonds and activation barriers, are DFT methods based on the wB97 functional [39]. Since the wB97XD functional is implemented in Gaussian16 program, it was used for further calculations of the activation energy because of its ability to perform calculations in the computational centre. Moreover, obtained activation energy values are closest to the reference method. The calculation of the B97-3c method, which is implemented in Orca software and is more than 10 times faster than the wB97XD method, was also added to all studied reactions for comparison.

The most time-consuming computational steps are the initial optimization of the reaction pathways and the calculation of the Hessian of the TS structures. In many cases, they can be performed at a much faster level using GFN2-xTB methods. For comparison, the computation (i5-7300H) of the Hessian on the geometrically optimized TS of HBR[4]A structure by the GFN2-xTB and B97-3c methods takes 10 min and 665 min, respectively. Surprisingly consistent values of the imaginary modes (*i*) for TS HBR[4]A were obtained, which are *i* = 338.77 cm^−1^ (GFN2-xTB) and *i* = 330.21 cm^−1^ (B97-3c), respectively. Owing to the very good compatibility between the imaginary modes and calculated activation energies for the generation reaction of *o-*QMR[4]A from HBR[4]A on GFN2-xTB and B97-3c geometries, further calculations for all other reactions were carried out on GFN2-xTB geometries.

Table 2 presents the collective data of reaction pathway calculations for HBR[4]A and ABR[4]A and their associated activation energies. Column two shows imaginary modes (*i*) for TS, while column three presents distances between the carbon atom of the benzyl group and the oxygen atom of the leaving group in TS. In the next columns, the activation energies calculated by the GFN2-xTB method and by DFT methods on GFN2-xTB geometries are shown.

**Table 2.** Summarization of the imaginary frequencies (*i*) and the distances between the carbon atom of the benzyl group and the oxygen atom in TS, as well as the activation energies of the formation of *o*-QMR[4]A from ABR[4]A and HBR[4]A ($\Delta E_{a}^{F}$) and the activation energies of the backward reaction ($\Delta E_{a}^{B}$).

R	imaginary mode (*i*)	distance H_2_C**..**OR	**GFN2-xTB** (kcal mol^−1^)		**B97-3c** (kcal mol^−1^)		**wB97XD** (kcal mol^−1^)	
	(cm^−1^)	(A)	$\Delta E_{a}^{F}$	$\Delta E_{a}^{B}$	$\Delta E_{a}^{F}$	$\Delta E_{a}^{B}$	$\Delta E_{a}^{F}$	$\Delta E_{a}^{B}$
H	*i*338.77	1.981	40.63	7.38	22.26	9.64	32.29	13.92
Me	*i*255.66	2.027	41.29	5.12	21.86	6.18	31.58	10.38
Et	*i*228.21	2.047	40.67	4.10	20.59	5.44	30.12	9.33
nPro	*i*238.16	2.029	40.55	3.56	20.50	6.20	30.05	10.40
iPro	*i*205.37	2.046	39.45	3.72	20.32	5.38	29.92	9.20
nBut	*i*237.69	2.027	40.53	4.56	20.52	6.28	29.87	10.22
tBut	*i*218.17	2.017	39.89	5.19	21.63	9.06	30.82	12.71

The calculated imaginary frequencies (*i)* for TS ABR[4]A have similar values and are in the range of *i* = 205–255 cm^−1^. A larger value for the imaginary frequency (reaction coordinate) *i* = 338 cm^−1^ was obtained for HBR[4]A. This is owing to the stronger bond between the carbon of the benzyl group and the oxygen atom of the hydroxyl group (PhH_2_C**-**OH), which is also reflected in the smallest distance of these atoms (1.981 A) in TS. For the remaining ABR[4]A, the distances between these atoms are very similar and are in the range of 2.017–2.047 A. This translates into a higher activation energy for the formation of *o*-QMR[4]A from HBR[4]A than the formation of *o*-QMR[4]A from ABR[4]A. Analysis of the calculated activation energies for these derivatives indicates that the highest values of forward (F) and backward (B) reaction activation energies were obtained for HBR[4]A for each of the calculation methods. The higher activation energy of $\Delta E_{a}^{F}$ is probably owing to the stronger carbon–oxygen bond for this derivative. On the other hand, in the case of the activation energy of the backward reaction $\Delta E_{a}^{B}$, this fact should be associated with the formation of a stronger hydrogen bond of the water molecule with the oxygen from the carbonyl group of the *o*-quinomethide unit in the formed product. The higher activation energy of the backward reaction in the case of t-butoxy benzyl derivative of resorcin[4]arene compared to other ABR[4]A is most likely owing to the steric hindrance of the t-butoxy group. In general, the values of the calculated activation energies for the formation of *o*-QMR[4]A from ABR[4]A are very close and are about 30 kcal mol^−1^. This is consistent with experimental observations showing that any of these derivatives can be used to generate *o*-QMR[4]A, especially methoxy and ethoxybenzyl derivatives of resorcin[4]arene, but also HBR[4]A [40].

Each of the theoretical methods used for the calculations reproduces general trends in the magnitude of the activation energy. The activation energies of *o*-QMR[4]A generation calculated by the GFN2-xTB method are overestimated by 10–20 kcal mol^−1^, while the activation energies of the backward reactions are underestimated by 3–7 kcal mol^−1^ compared to the DFT methods. This is not particularly unusual because the GFN2-xTB method is not parameterized for reaction energy. From the comparison of the DFT methods from the first (B97-3c) and third (wB97XD) rungs of Jacob's ladder, it can be seen that the fast B97-3c method underestimates the activation energies of *o*-QMR[4]A formation relative to the wB97XD method by about 10 kcal mol^−1^ and by about 4 kcal mol^−1^ for the backward reaction, respectively.

**Figure 2.**
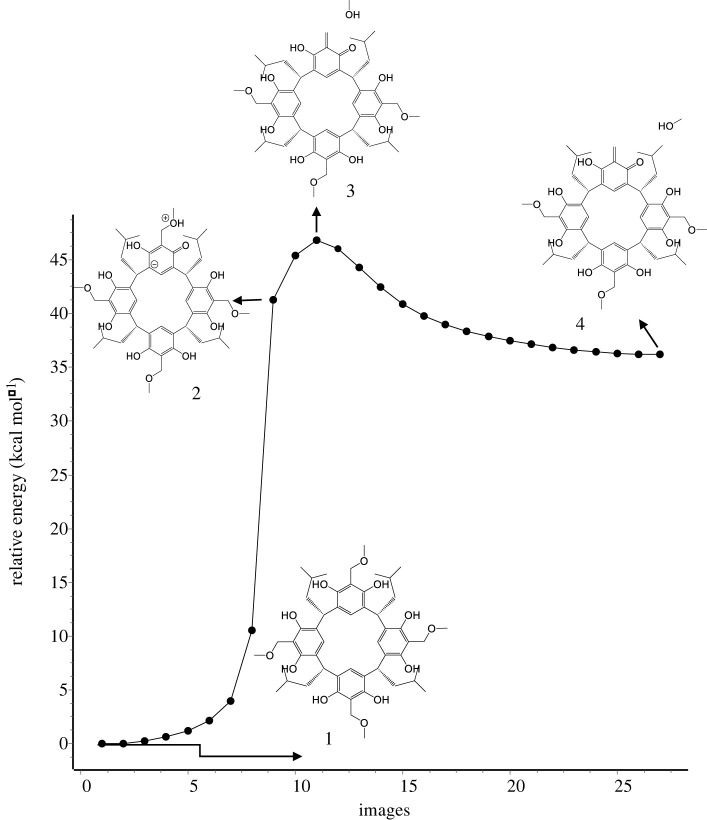
The reaction pathway trajectory of *o*-QMR[4]A formation from methoxybenzyl derivative of resorcin[4]arene calculated by the NEB-TS IRC/GFN2-xTB method, along with selected structures showing the reaction mechanism.

The mechanism of *o-*QMR[4]A generation from ABR[4]A is shown in figure 2, using the methoxybenzyl derivative of resorcin[4]arene as an example. Figure 2 shows the dependence of the reaction pathway trajectory of *o*-QMR[4]A formation, calculated by the NEB-TS IRC method, along with selected structures illustrating the reaction mechanism. For all other ABRs[4]A, reaction pathway analysis indicates that the reaction mechanism for *o*-QMR[4]A formation is very similar. The methoxybenzyl derivative of resorcin[4]arene was used, among others, in Michael and cycloaddition reactions, which was described in [11,14–16].

The crystal structure of the methoxybenzyl derivative of resorcin[4]arene indicates a strong intramolecular hydrogen bond between the methoxy group of resorcin[4]arene and the hydroxyl group [9]. The high acidity of hydroxyl groups in the methoxybenzyl derivative of resorcin[4]arene is also indicated by PM6-D3H4 calculations (pKa_1_ = 4.19) using Mopac2016 [41]. This has consequences in the mechanism of the reaction, as proton transfer to the methoxy group in the resorcin[4]arene (structure 2) occurs before TS is reached. Only in the next step, the departure of the methanol molecule occurs with the formation of TS *o-*QMR[4]A (structure 3). In TS, one of the hydrogens of the methylene group in *o*-QMR[4]A is turned with respect to the plane of the benzene ring by an angle of −31.5° (dihedral angle C12-C13-C6-H119). In a further step, it is flattened to form the *o*-QMR[4]A product (structure 4). The similarity of the TS structure (3) to the P structure (4) (owing to the small difference in their energies) is consistent with Hammond's postulate [42].

### 2.3. Reactivity of *o*-QMR[4]A with H-nucleophiles and the reaction mechanism involving them

**Figure 3.**
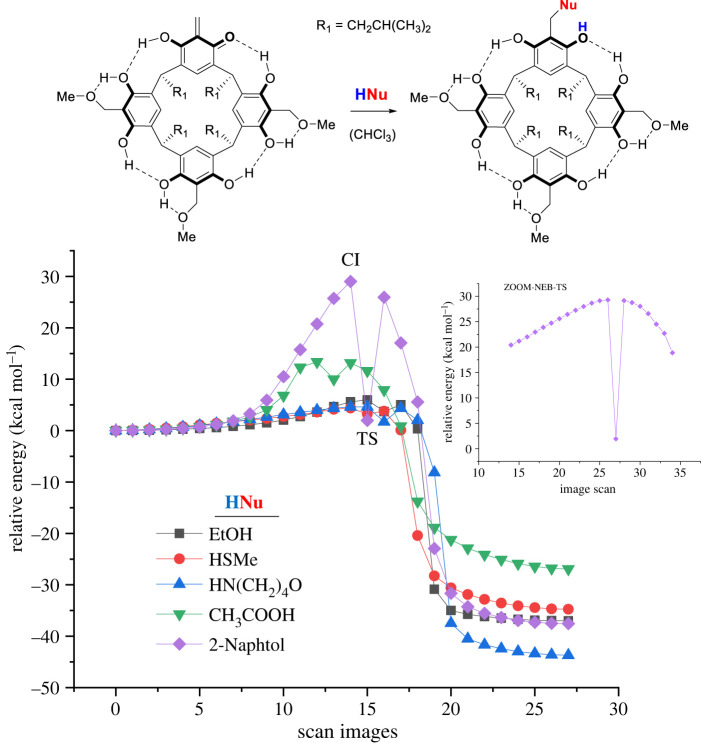
Relative energy changes in function of number of scans of *o*-QMR[4]A formation pathways with selected nucleophiles generated while using the NEB-TS IRC/GFN2-xTB method. The upper right corner shows a scan fragment for 2-naphthol generated by the ZOOM-NEB-TS IRC/GFN2-xTB method.

The next step was to theoretically investigate the reactivity of *o*-QMR[4]A generated from a methoxybenzyl derivative of resorcin[4]arene with selected HNu of varying nucleophilicity. The reaction pathways by the NEB-TS IRC/GFN2-xTB method were investigated against ethanol, thiomethanol, morpholine, acetic acid and 2-naphthol in chloroform. Besides thiomethanol, reaction products with the listed nucleophiles are known in the literature. However, thiomethanol was included in the calculation to predict the reactivity of this group of compounds as nucleophiles when reacted with *o*-QMR[4]A. The calculation procedure was identical to that described previously. The reaction pathways of *o*-QMR[4]A with nucleophiles along with the reaction scheme are shown in figure 3. A rather unexpected reaction pathway was obtained for 2-naphthol, for which TS was located much lower than for the CI scan on the relative energy scale. In view of the above, an additional scan was performed for this case using the ZOOM-NEB-TS IRC command in Orca 4.2.1, which more accurately scans the potential energy around the TS. A fragment of this scan for 2-naphthol is included in figure 3.

The data on the imaginary modes (*i*) of TS, the distance between the methine carbon of *o*-QMR[4]A and the nucleophile atom, and the values of the activation energies of the *o*-QMR[4]A-nucleophile reactions and backward reactions calculated by GFN2-xTB and DFT methods are summarized in table 3. The imaginary modes (*i*) of TS (except for TS involving morpholine) are in the range of 233–170 cm^−1^. In case of TS with the morpholine molecule, the calculated imaginary frequency is 25.63 cm^−1^. As this frequency seemed to be too low, additional calculations were made at the level of the B97-3c geometry. The calculated value of imaginary frequency *i* = 20.75 cm^−1^ is consistent with the value obtained at the GFN2-xTB geometry level.

**Table 3.** Imaginary frequencies (*i*), distances of the carbon atom of the methine group in *o-*QMR[4]A from the nucleophilic atom in TS and also the activation energies of the reaction of *o*-QMR[4]A with nucleophiles ($\Delta E_{a}^{F}$) and the activation energies of the backward reaction ($\Delta E_{a}^{B}$). (Calculations were performed on GFN2-xTB geometries.)

HNu	imaginary mode (*i*)	distance H_2_C**..**Nu	**GFN2-xTB** (kcal mol^−1^)		**B97-3c** (kcal mol^−1^)		**wB97XD** (kcal mol^−1^)	
	(cm^−1^)	(A)	$\Delta E_{a}^{F}$	$\Delta E_{a}^{B}$	$\Delta E_{a}^{F}$	$\Delta E_{a}^{B}$	$\Delta E_{a}^{F}$	$\Delta E_{a}^{B}$
EtOH	*i*232.65	2.043	3.70	40.70	6.16	20.53	10.22	30.14
MeSH	*i*207.64	3.826	3.23	37.98	2,58	28,23	2.53	32.11
morpholine	*i*25.63	2.827	1.70	45.39	1.75	25.13	3.33	31.58
CH_3_COOH	*i*217.45	2.416	10.00	36,90	15.10	22,38	19.30	31.35
2-naphthol	*i*170.33	2.346	1.97	39.52	3,30	23,48	9.11	34.74

The distances in TS vary depending on the type of nucleophile molecule. The lowest value (2.04 A) is obtained for the reaction with the ethanol molecule, followed by the 2-naphthol molecule (2.35 A) and the acetic acid molecule (2.42 A). For the reaction with morpholine molecule, this distance is already clearly larger and amounts to 2.83 A, while for thiomethanol molecule it is relatively large and amounts to 3.83 A. It can be connected in general with the concept of hardness-softness of the nucleophile atom [43,44] in the molecule interacting with the carbon atom of the methine group in *o-*QMR[4]A.

The calculated activation energies shown in table 3 are quite strongly divergent depending on the calculation method used. The values obtained while using the GFN2-xTB method significantly differ from the values generated by the DFT methods and differ from the values obtained by the wB97XD method by 1.63–9.30 kcal mol^−1^ for $\Delta E_{a}^{F}$ and 4.78–10.56 kcal mol^−1^ for $\Delta E_{a}^{B}$, respectively, depending on nucleophile type. The differences in calculated activation energies by the DFT methods are in the range of 0.05–5.8 kcal mol^−1^ for $\Delta E_{a}^{F}$, while for $\Delta E_{a}^{B}$ they reach 11 kcal mol^−1^. However, they are within the accuracy of the DFT methods, as demonstrated for reaction energies and energy barrier heights tested for large molecular systems (GMTKN55 database) [45]. The data discussed here therefore show with how much caution the obtained activation energy values should be used and how strongly they depend on the calculation method used. However, it is worth noting that the DFT methods used capture the trend of activation energy changes in *o*-QMR[4]A reactions with nucleophile molecules.

**Figure 4.**
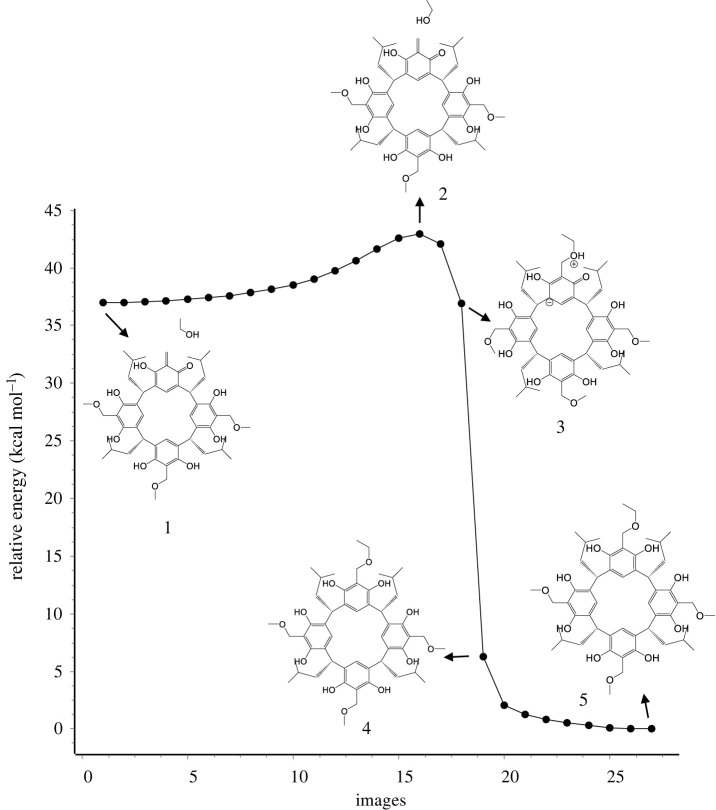
Trajectory of the reaction pathway of o-QMR[4]A with ethanol calculated by the NEB-TS IRC/GFN2-xTB method along with selected structures showing the reaction mechanism.

Analysis of the reaction pathways of *o*-QMR[4]A with selected nucleophilic molecules shows that the reaction mechanisms leading to the product molecules are slightly different. Figure 4 shows the trajectory of the reaction pathway of *o*-QMR[4]A with ethanol calculated by the NEB-TS IRC/GFN2-xTB method along with selected structures showing the reaction mechanism. As the reaction proceeds, the *o*-QBR[4]A molecule bound to ethanol via hydrogen bonding (structure 1) undergoes a rearrangement that involves turning the methine group in the *o-*quinomethide unit with a simultaneous approach of the oxygen atom of the ethanol molecule to the methine carbon atom with the formation of TS (structure 2). In the next step, their further closing takes place, so that in the next step a bond (C-O) of the methine carbon and the oxygen atom of the ethanol molecule is formed (structure 3). Intramolecular proton transfer from the ethoxyoxonium group to the carbonyl group of the *o*-quinomethide unit leads to restoration of the aromaticity of the resorcinol ring with simultaneous stabilization of the energy of the molecule (structure 4). Subsequent scans show only energy changes leading to the energy minimum of the formed product (structure 5).

**Figure 5.**
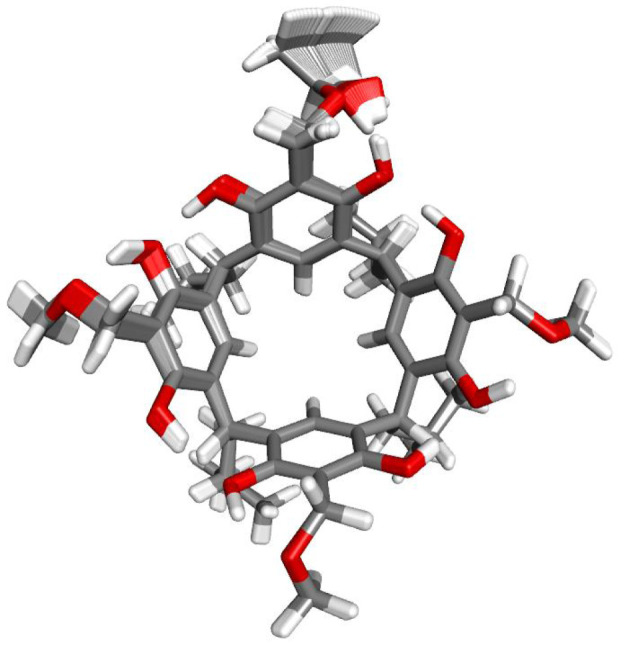
Overlay scans of the *o*-QMR[4]A reaction pathway with ethanol generated by the NEB-TS IRC/GFN2-xTB method.

Overlaying all the reaction path scans shows that the ethanol addition reaction to one *o*-QMR[4] unit is a dynamic process and affects the structure of other units in the resorcin[4]arene. A particularly strong effect is seen on the structure of the neighbouring resorcinol unit through hydrogen bonding of the hydroxyl group of the *o*-quinomethide unit (figure 5) with the neighbouring resorcinol unit. The smallest structural changes are observed for the opposite resorcinol unit in the resorcin[4]arene.

**Figure 6.**
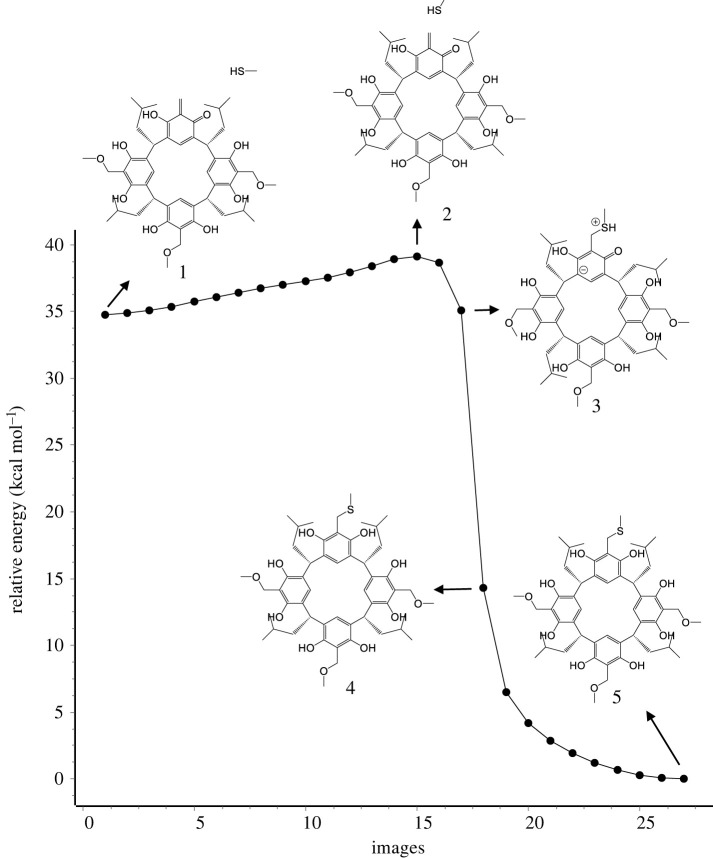
Trajectory of the reaction pathway of *o*-QMR[4]A with methanethiol calculated by the NEB-TS/GFN2-xTB method along with selected structures showing the reaction mechanism.

A similar reaction mechanism is observed for the addition of morpholine and 2-naphthol to *o*-QBR[4]A. However, for thiomethanol and especially acetic acid, the mechanism is a little bit different. In the case of thiomethanol (figure 6), after reaching TS (structure 2), the subsequent steps are followed by a shift of the hydrogen atom forward to the oxygen of the carbonyl group until it is shared between the sulfur and oxygen atoms (structure 3). In the next step, its full transfer to the oxygen atom occurs with simultaneous bond formation between carbon and sulfur atoms along with aromaticity recovery (structure 4). In further steps, the energy stabilization of the formed sulfonic resorcin[4]arene derivative (structure 5) takes place.

**Figure 7.**
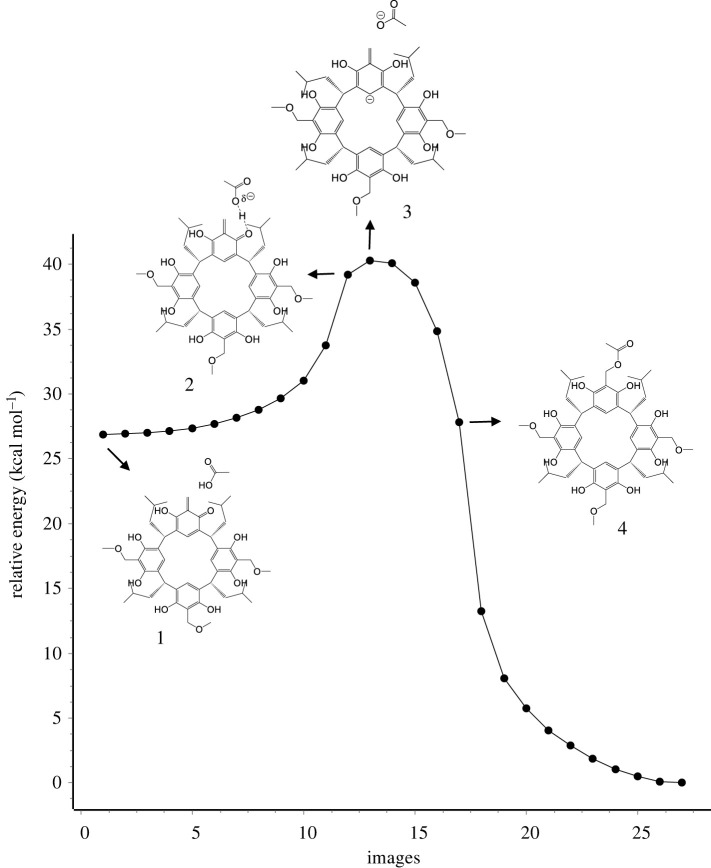
Trajectory of the reaction pathway of *o-*QMR[4]A with acetic acid calculated by the NEB-TS/GFN2-xTB method along with selected structures showing the reaction mechanism.

The reaction with acetic acid is characterized by a different profile of the reaction course (figure 7). The high proton acidity of the carboxyl group results in a process of proton transfer to the carbonyl oxygen of the *o*-QMR[4]A molecule even before the TS is reached, until it is shared between the oxygen atoms (structure 2). In the next step, TS is reached with the transfer of a proton to a carbonyl oxygen atom with the formation of the benzyl carbocation *o*-QBR[4]A (structure 3). The formation of the acetate anion increases the nucleophilicity of the oxygen atoms and in the next few steps the acetate anion is attached via the oxygen atom to the benzyl cation (structure 4) with the formation of a mono-acetate derivative of resorcin[4]arene.

**Figure 8.**
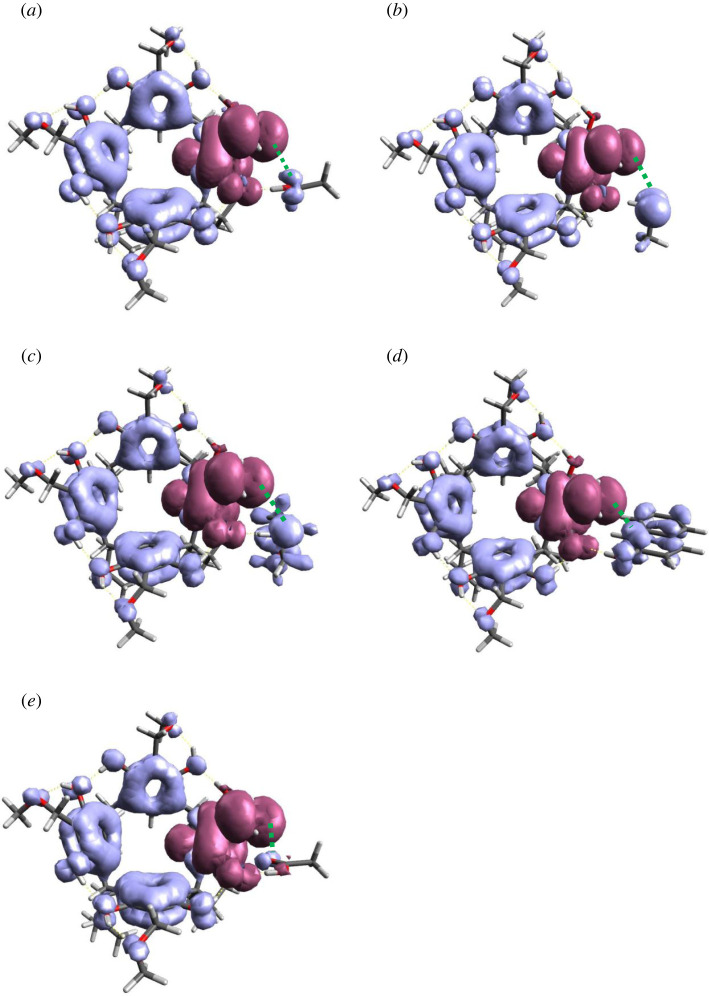
Isosurfaces of orbital-weighted dual descriptor (Δ*f_w_*) of reactants (R) in the *o*-QBR[4]A reaction with (*a*) ethanol, (*b*) methanethiol, (*c*) morpholine, (*d*) 2-naphthol and (*e*) acetic acid.

Fukui reactivity coefficients (*f^+^* and *f^−^*) are often used to explain reaction mechanisms [46]. High values of *f^+^* or *f^−^* indicate the most favourable sites for accepting or donating electrons, characterizing sites of high electrophilicity or nucleophilicity, respectively. As shown in [47], a better approach to describe the reactivity of molecules is to use orbital-weighted Fukui functions ($f_{w}^{+}$ and $f_{w}^{-}$) and orbital-weighted dual descriptor (Δ*f_w_*). For this purpose, a dual descriptor was quantitatively calculated based on population analysis techniques using the Multiwfn program [48]. The obtained values for the energy optimized (SPE in B97XD) reaction reactants (R) were visualized by the corresponding isosurfaces shown in figure 8.

The grey and maroon isosurfaces represent the nucleophilic and electrophilic fragments of the reacting molecules, respectively. The green dashed line in this figure indicates the interacting fragments (atoms) of the reacting molecules. Large isosurfaces for morpholine and thiomethanol (figure 8*b*,*c*), associated with large values of weighted descriptors Δ*f_w_* indicate their high nucleophilicity and strong interaction with methine carbon *o*-QBR[4]A. This is in agreement with the calculated backward reaction activation barriers, which are much lower for these nucleophiles compared to the others (table 3). This is also confirmed by experimental data showing that the reactions of *o-*quinomethide derivatives of phenols, owing to their high reactivity, proceed practically barrier-free with nucleophiles such as amines or thiols [48].

### 2.4. Computational details

Calculations for the optimization of reactant and product geometries and scanning of the potential energy of the products were performed using the semiempirical GFN2-xTB method using the xTB 6.33 package [49]. The NEB and IRC methods present in the Orca 4.2.1 package with an implementation to support xTB method calculations were used to search for TS. SPE calculations with DFT methods using the functionals B97-3c, revPBE-D4, B97M-D4 B97M-V, B3LYP-D4, wB97M-D3BJ were performed using the Orca 4.2.1 package. The def2-TZVP functional base was used for all these calculations, except for method B97-3c, in which the def2-mTZVP base was implemented. For all functionals, dispersion corrections D3BJ [50] or D4 [51] were applied if that was possible. In case of the B97M-D4 method, the D4 parameters described in [35] were used. All GGA and meta-GGA functionals in Orca were also treated with the RI-J approximation, whereas hybrids functionals were treated with the chain-of-sphere approximation to evaluate exchange integrals (RIJCOSX). Orca's default settings were used in the latter case. All the above calculations were performed on a laptop equipped with a 4-core i5-7300H processor. SPE calculations using the wB97XD method for all R, P and TS of the reactions studied were calculated using the Gaussian16 package. The calculations were carried out in a computer centre (Wroclaw Network and Computer Centre, Poland) using 12 processors. Isosurfaces of the orbitally weighted dual descriptor (Δ*f_w_*) for the reactants were performed using the Multiwfn 3.8 package and its visualization using the Avogadro 1.2 program [52].

## 3. Conclusion

In this paper, the authors have calculated the activation energies of *o-*QMR[4]A generated from HBR[4]A and ABR[4]A as well as activation energies of backward reactions at different levels of quantum-mechanical methods: semiempirical (GFN2-xTB) and DFT (B97-3c, wB97XD). Similar estimates were made for the reaction of *o*-QMR[4]A with HNu nucleophiles. Using HBR[4]A as an example, activation energy calculations ($\Delta E_{a}^{F}$ and $\Delta E_{a}^{B}$) were performed with selected (Jacob's ladder based) DFT methods on GFN2-xTB and B97-3c geometries. Very good agreement was found between the results obtained, which allowed further calculations based only on GFN2-xTB geometries. This is a very important finding from a computational cost perspective. DFT calculations on GFN-xTB geometries provide insight into the reaction mechanisms of compounds with a large number of atoms in the molecule.

Based on the obtained data for all reactions, it can be seen that the GFN2-xTB method overestimates by about 10 kcal mol^−1^ the calculated activation energies, while the B97-3c method underestimates by about 4–10 kcal mol^−1^ compared to the wB97XD method. The activation energies of the generation of *o*-QMR[4]A from HBR[4]A, calculated using the wB97XD functional, are very similar at about 30 kcal mol^−1^, while the activation energies of the backward reactions depend to some extent on the substituent type and are in the range of 9–14 kcal mol^−1^. On the other hand, the activation energies for *o*-QMR[4]A reactions with nucleophiles are in the range of 2–20 kcal mol^−1^ depending on the type of nucleophile, while for backward reactions, they are in range of 30–35 kcal mol^−1^.

The reaction pathways calculated by the NEB-TS IRC method allowed the tracing of the discussed reaction mechanisms. In case of formation of o-QMR[4]A from HBR[4]A and ABR[4]A, the reaction mechanism is very similar and is based on initial protonation of the hydroxybenzyl group or the alkoxybenzyl derivative of resorcin[4]arene by the proton of the hydroxyl group of the resorcinol ring, followed by the departure of a water molecule or a molecule of the corresponding alcohol with the generation of *o*-QMR[4]A. The 1,4-Michael reactions of *o*-QMR[4]A with nucleophiles are characterized by different mechanisms depending on the type of nucleophile molecule. For nucleophiles such as ethanol, morpholine and 2-naphthol, a clearly two-step mechanism can be observed. In the first step, the nucleophilic atom of the nucleophilic molecule is attached to the methine carbon of the *o*-QMR[4]A, followed by proton transfer from the nucleophilic molecule to the carbonyl oxygen in the second step with simultaneous restoration of aromaticity and energy stabilization of the formed product. In the case of the methanethiol molecule, a thiomethanol proton is shared between the sulfur atom and the carbonyl oxygen atom of o-QMR[4]A in the initial step. In the next step, synchronous attachment of the sulfur atom to the methine carbon occurs with a simultaneous proton transfer to the carbonyl oxygen atom in *o*-QMR[4]A. The reaction with acetic acid, on the other hand, occurs first by the formation of a benzyl carbocation and then its reaction with the oxygen atom of the acetate anion.

The reactivity of *o*-QMR[4]A with nucleophiles was discussed and visualized based on the calculated orbital-weighted dual Fukui descriptor, which describes well the electrophilic and nucleophilic sites of the molecule.

## Data Availability

All data necessary to reproduce the calculations presented in the manuscript are provided in the electronic supplementary material [53].
